# Classification of human coronary atherosclerotic plaques with T1, T2 and Ultrashort TE MRI

**DOI:** 10.1186/1532-429X-14-S1-P135

**Published:** 2012-02-01

**Authors:** Mihaly Karolyi, Harald Seifarth, Gary Liew, Christopher L Schlett, Pal Maurovich-Horvat, Guangping Dai, Shuning Huang, Craig J Goergen, Udo Hoffmann, David E Sosnovik

**Affiliations:** 1 Massachusetts General Hospital, Charlestown, MA, USA; 2 Massachusetts General Hospital, Harvard Medical School, Boston, MA, USA

## Summary

Multicontrast MRI with T1, T2 and Ultrashort TE (UTE) sequences is used to image atherosclerotic plaque in human coronary arteries. MRI classification of the plaques is compared with their histological classification and found to correlate extremely well. The addition of UTE MRI adds significant value to the imaging of human coronary artery plaque by MRI.

## Background

The differentiation of atherosclerotic plaque components in the carotid arteries with MRI has been successfully demonstrated. The detection of plaque calcification by MRI, however has been challenging. In addition, few studies have evaluated the ability of MRI to characterize atherosclerotic plaques in human coronary arteries (1). Here we use a combination of T1, T2 and ultrashort TE (UTE) MRI to evaluate atherosclerotic plaques in fixed post-mortem human coronary arteries. We hypothesized that the addition of UTE to T1and T2 weighted MRI would allow both calcified and lipid rich plaques to be accurately detected and distinguished from one another.

## Methods

Twenty eight plaques from human donor hearts with proven coronary artery disease were imaged on 9.4T horizontal bore MRI scanner (Biospec, Bruker). The specimens were imaged with a T1W 3D FLASH sequence (250 um isotropic resolution, TR/TE 30/2.5ms), a T2W Rare sequence (slice thickness 0.4 mm, in plane resolution 0.156mm, TR/TE 3000/40ms), and an UTE sequence (200-300um resolution, TE of 20us). Plaques showing selective hypointensity on T2W MRI were classified as lipid rich. Areas of hypointesnity on the T1W images but not the UTE images were classified as calcified. After MRI, the plaques were sectioned for histological characterization with a pentachrome stain, which was used as the gold standard readout. The histological sections were digitized and co-registered with the MR images for analysis.

## Results

**Figure 1:**
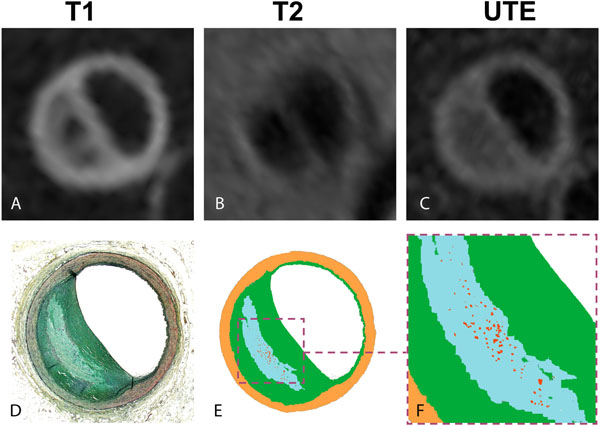
T1, T2 and UTE images of a calcified lipid rich necrotic plaque. (A) T1 weighting reveals a large plaque with two focal areas of profound hypointensity. These areas are isointense with the rest of the plaque on the T2 (B) and UTE (C) images, consistent with foci of calcification. (B) profound hypointensity is seen in the plaque on the T2 weighted image, consistent with a lipid-rich necrotic core. (D) Pentachrome staining of the plaque section correlates extremely well with the MRI. (E, F) Segmentation of the histological section showing the lipd-rich necrotic core (light blue) and foci of calcification (red).

**Figure 2:**
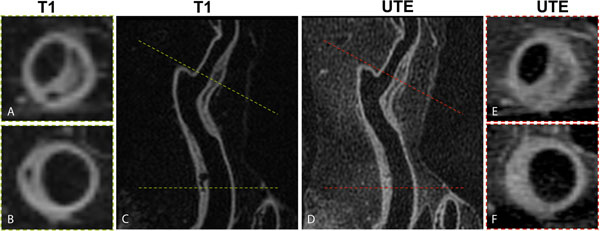
T1 weighted and UTE MRI of a coronary artery with large fibrocalcific plaques. (A, B) T1W MRI at two cross-sections along the coronary artery. (C) T1 weighted multiplanar reformat of the vessel. (D) UTE multiplanar reformat, and (E, F) UTE cross sections of the vessel. Two large plaques are seen along the coronary artery in the multiplanar reformats. Foci of profound T1 hypointensity, which appear isointense on the UTE images, are seen in these plaques consistent with plaque calcification.

Plaque classification by MRI was performed by 4 readers who were blinded to the histological plaque classification. The sensitivity and specificity of MRI for the detection of plaque calcification were 100.0% and 90.0% respectively. The sensitivity and specificity of MRI for the detection of lipid rich necrotic cores were 90.0%, 75.0%, respectively. Good inter-observer agreement was found for plaque classification by MRI (κ = 0.7829, p<0.0001). More importantly, MRI categorization of coronary artery plaques agreed well with the histological classification (weighted κ =0.6945, p<0.0001). An example of a lipid-rich necrotic plaque with calcification is shown in Figure 1. A fibrocalcific coronary artery plaque is shown in Figure 2.

## Conclusions

MRI with T1, T2 and UTE contrast is able to accurately classify atherosclerotic plaques in human coronary arteries. This underscores the need to develop techniques to image the coronary artery wall in patients in vivo.

## Funding

R01 HL093038 and NCRR P41RR14075

## References

[B1] Itskovich MRM 2004

